# Hazardous alcohol use and HIV indicators in six African countries: results from the Population‐based HIV Impact Assessments, 2015–2017

**DOI:** 10.1002/jia2.26029

**Published:** 2022-11-21

**Authors:** Gregory C. Chang, Christine A. West, Evelyn Kim, Andrew J. Low, Kathryn E. Lancaster, Stephanie S. Behel, Steven Y. Hong, Leigh Ann Miller, Rachel Silver, George S. Mgomella, Jennifer Imaa, Werner M. Maokola, Thomas Carpino, Gili Hrusa, Rachel M. Bray, Annie Mwila, Godfrey Musuka, Christopher O'Connell, Stephen McCracken, Andrew C. Voetsch

**Affiliations:** ^1^ Division of Global HIV and TB Center for Global Health US Centers for Disease Control and Prevention Atlanta Georgia USA; ^2^ PHI/CDC Global Health Fellowship Program Oakland California USA; ^3^ Division of Global HIV and TB Center for Global Health US Centers for Disease Control and Prevention Lilongwe Malawi; ^4^ ICAP at Columbia University New York New York USA; ^5^ Division of Epidemiology College of Public Health The Ohio State University Columbus Ohio USA; ^6^ Division of Global HIV and TB Center for Global Health US Centers for Disease Control and Prevention Windhoek Namibia; ^7^ Division of Global HIV and TB Center for Global Health US Centers for Disease Control and Prevention Dar es Salaam Tanzania; ^8^ National AIDS Control Programme Ministry of Health Community Development Gender Elderly and Children Dar es Salaam Tanzania; ^9^ Division of Global HIV and TB Center for Global Health US Centers for Disease Control and Prevention Lusaka Zambia; ^10^ ICAP at Columbia University Harare Zimbabwe; ^11^ Center for Substance Abuse Prevention Substance Abuse and Mental Health Services Administration Rockville Maryland USA

**Keywords:** HIV care continuum, PHIA, hazardous alcohol use, hazardous drinking, UNAIDS 90‐90‐90, HIV epidemiology

## Abstract

**Introduction:**

Hazardous alcohol use (HAU), defined as a pattern of alcohol consumption that increases the risk of harmful consequences for the user or others, is associated with an elevated risk of human immunodeficiency virus (HIV) infection and poor health outcomes. We describe the association between people living with HIV (PLHIV) who report HAU and key HIV indicators. Gaps in current literature in estimating HAU on HIV outcomes at the regional level of Eastern and Southern Africa still exist and our analysis aims to address this issue.

**Methods:**

We used weighted pooled data (2015–2017) from the nationally representative Population‐based HIV Impact Assessments among adults who provided written consent aged 18–59 years from Eswatini, Malawi, Namibia, Tanzania, Zambia and Zimbabwe. We estimated differences in the prevalence of HIV infection and The Joint United Nations Programme on HIV and AIDS (UNAIDS) 90‐90‐90 indicators between PLHIV by HAU status using log‐binomial regression, stratified by sex. HAU was determined using the Alcohol Use Identification Test—Consumption.

**Results:**

Among the 9755 women and 4444 men who tested HIV positive, 6.6% of women and 21.8% of men engaged in HAU. Women who reported HAU were more likely to be HIV positive (adjusted prevalence ratio [aPR] = 1.31, 95% CI: 1.18–1.46) compared to those who did not report HAU. For the UNAIDS 90‐90‐90 targets, women who engaged in HAU were more likely to be unaware of their HIV‐positive status (aPR = 1.22, 95% CI: 1.01–1.47) and not on antiretroviral therapy (ART) (aPR = 1.73, 95% CI: 1.26–2.37). Men who engaged in HAU were more likely to be unaware of their HIV‐positive status (aPR = 1.56, 95% CI 1.39–1.76) and not on ART (aPR = 1.72, 95% CI: 1.30–2.29). No difference in viral load suppression, defined as <1000 copies/ml of HIV RNA, was seen by sex.

**Conclusions:**

PLHIV who engage in HAU were more likely to have suboptimal outcomes along the HIV care continuum when compared to those who did not engage in HAU. Targeted interventions, such as alcohol screening for HAU in HIV testing and treatment settings and HIV prevention efforts in alcohol‐based venues, may help countries reach HIV epidemic control by 2030.

## 1 INTRODUCTION

As of 2020, Eastern and Southern Africa (ESA) account for approximately 20.7 million people living with human immunodeficiency virus (PLHIV), resulting in 55% of all infections globally and 44% of all new global infections occurring in this region [1, 2]. Despite a 49% decrease in HIV‐related deaths since 2010–2020, it is estimated that over 310,000 individuals succumbed to HIV‐related illness, as well as 600,000 new infections occurred in 2020 [1]. To reach HIV epidemic control, the Joint United Nations Programme on HIV/AIDS (UNAIDS) has set its ambitious 95‐95‐95 “Fast‐Track” targets, which entails getting 95% of all PLHIV to know their HIV status, 95% of people who know their status to receive HIV treatment and 95% of people on HIV treatment to have viral load suppression (VLS) by 2030 [3]. The 2030 target had an interim 90‐90‐90 target to be reached by 2020, a metric that was not met across most of ESA [1, 2]. As of 2022, neither a cure nor effective vaccine exists for HIV; therefore, lifelong treatment on antiretroviral therapy (ART) remains the gold standard for disease management [4].

Hazardous alcohol use (HAU), defined as a pattern of alcohol consumption that increases the risk of harmful consequences for the user or others, is associated with both elevated risk of acquiring HIV and poor health outcomes among PLHIV [5, 6, 7, 8]. Alcohol consumption is associated with an increased likelihood of engaging in behaviours that elevate the risk of HIV acquisition, such as decreased sexual inhibition, acts of condomless sex and multiple sexual partners [6, 7, 8, 9]. In addition, excessive alcohol consumption can result in deferred testing for HIV infection, delayed linkage to ART initiation for newly diagnosed PLHIV and lower treatment adherence [7, 10]. Individuals who engaged in hazardous drinking may skip ART doses due to misperceptions of possible alcohol and ART interactions or forget to take their treatment or neglect to refill their prescriptions due to intoxication [11, 12, 13, 14]. Furthermore, in a systematic review that pooled estimates of alcohol use on ART non‐adherence across 32 studies, individuals who used alcohol had two times the odds of ART non‐adherence and two and a half times the odds of having unsuppressed HIV viral load compared to those who did not use alcohol [15].

Poor ART adherence can lead to an overall higher viral load, and can lead to the emergence of ART‐resistant viral strains, progression to acquired immunodeficiency syndrome (AIDS) and AIDS‐related death [16]. Undiagnosed, untreated or non‐adherent PLHIV also have a higher likelihood of onward sexual transmission [17, 18, 19, 20]. Therefore, focusing on risk factors, such as hazardous drinking, can benefit primary and secondary HIV prevention efforts, in addition to improving health outcomes for PLHIV.

Previous studies have examined the relationship between alcohol use and various HIV outcomes; however, there remains a lack of population‐based estimates of the impact of HAU on the HIV care cascade in ESA, the region with the highest burden of HIV. We examined the relationship between HAU and key HIV indicators using data from the Population‐based HIV Impact Assessments (PHIA) surveys in six countries in ESA. These nationally representative surveys include PLHIV who are not captured in health facility data and provide broad generalizability due to the population‐based sampling design. In addition, our study sample size was large enough to assess HAU and HIV outcomes adjusted for demographic covariates stratified by sex [21, 22, 23]. The objective of the analysis is to estimate the effects of HAU on the achievement of the component UNAIDS 90‐90‐90 targets. We also aim to identify gaps in awareness of HIV‐positive status, ART treatment and VLS among adults 18–59 years old by sex and alcohol consumption behaviour.

## 2 METHODS

### 2.1 Study design and participants

Our dataset was derived from six PHIA surveys that asked alcohol‐related questions as part of the core questionnaire. The surveys that were included were Eswatini (2016–2017), Malawi (2015–2016), Namibia (2017), Tanzania (2016–2017), Zambia (2016) and Zimbabwe (2015–2016). These six surveys were selected due to the data being publicly available upon request from ICAP at the time of the analysis (2015–2017). The PHIA methods have been previously described [24, 25]. Briefly, PHIA surveys are cross‐sectional nationally representative, household‐based surveys conducted among adults aged 15 years and older, as well as children 0–14 years in some countries [25]. The main objectives of the PHIAs are to measure national HIV prevalence and VLS, nationally and sub‐nationally, to assess the impact of HIV treatment and prevention programmes in each country. Participants for the survey, who provided written consent, undergo both an interview component, where participants answer a core set of questions that are comparable across all PHIA surveys, and a biomarker component. Alcohol‐related questions were included in a limited amount of PHIA surveys and also varied by country. For these surveys, the biomarker testing included in‐home HIV rapid testing in line with the respective country's national HIV rapid testing algorithm, point‐of‐care CD4 count, viral load count and antiretroviral (ARV) detection of drugs commonly used in each respective country.

We limited our analysis to participants aged 18–59 years based on the legal drinking age in the six surveys. Individuals who consented to both the individual interview and blood draw answered the alcohol‐related questions and had a final HIV status classification were considered eligible for our analysis.

### 2.2 Variable definition

Drinking status was based on the World Health Organization's (WHO) Alcohol Use Disorders Identification Test‐Consumption (AUDIT‐C), which identifies individuals who engage in HAU or have harmful patterns of alcohol consumption [5]. This tool is an abbreviated version of the full 10‐question AUDIT tool, which has traditionally been the gold standard for identifying individuals who engage in HAU. The AUDIT‐C tool is comprised of three questions scored on a scale of 1–12 points, where each question has five answers, and 0–4 points are attributed to each answer. Individuals who score ≥4 and ≥3 among men and women, respectively, are considered as screening positive for HAU. The AUDIT‐C tool has been validated in various settings and different racial/ethnic groups, and the results have been comparable to the full AUDIT [26, 27, 28, 29].

HIV‐specific outcomes that were analysed include HIV prevalence as well as the UNAIDS 90‐90‐90 targets. For the first 90 target, we analysed the proportion of participants who were aware of their HIV‐positive status; for the second 90 target, we analysed the proportion of those who were on ART among those aware of their HIV‐positive status, and for the third UNAIDS 90 target, the proportion of individuals who were on ART and had VLS. VLS was determined if the participant had less than 1000 HIV RNA copies per millilitre of blood. Individuals were considered aware of their HIV‐positive status if they either reported being positive or if ARVs were detected in their blood specimens. Likewise, ART status was determined by participants either reporting being on treatment at the time of the survey and/or had detectable ARVs in their blood. We also calculated the unconditional treatment status among all HIV‐positive individuals, which is defined as the proportion of individuals who were on ART regardless of their awareness of their HIV status. The unconditional VLS status among HIV‐positive participants was also calculated. This included the proportion of HIV‐positive individuals who were virally suppressed regardless of their treatment status.

### 2.3 Statistical analyses

We compared HIV prevalence and achievement of conditional and unconditional 90‐90‐90 targets between participants who reported hazardous and non‐HAU. Survey weights were applied to account for differences in sampling probabilities and to adjust for survey non‐response. Weighted prevalence estimates and 95% confidence intervals (CI) of demographic factors were calculated for those who reported hazardous versus non‐HAU by sex. Taylor series expansion was used to calculate variance estimations for the complex survey data and also accounts for a stratified, cluster sampling design. Survey‐weighted prevalence estimates were conducted in SAS version 9.4 (SAS Institute, Cary, North Carolina). We also calculated adjusted prevalence ratios (aPRs) comparing conditional and unconditional UNAIDS 90‐90‐90 targets between those who reported HAU versus non‐HAU in a log‐binomial regression model using SAS‐callable SUDAAN (RTI International, Research Triangle Park, North Carolina). The aPR model covariates were determined by bivariate analyses with a *p*‐value <0.10 in addition to previous literature. Model covariates include a place of residence (urban vs. rural), global wealth quintile, age, country and marital status. Because the prevalence of alcohol use is different between men and women in ESA, all analyses were stratified by sex [30, 31, 32].

### 2.4 Implementation and ethics

This project is supported by the United States President's Emergency Plan for AIDS Relief through the US Centers for Disease Control and Prevention (CDC) under the terms of cooperative agreements #U2GGH001271 and #U2GGH001226. Each survey was reviewed and approved by human subjects and Institutional Review Boards at the CDC, Columbia University, Westat and in the respective countries. All survey participants provided informed consent prior to survey participation.

## 3 RESULTS

**Figure 1:**
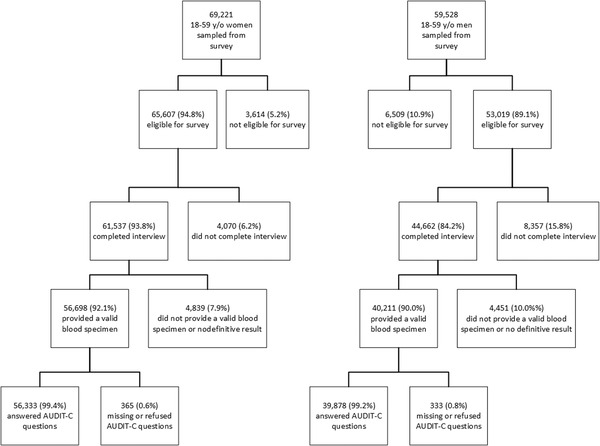
Flow chart of eligibility, interview completion, blood sampling and AUDIT‐C responses among 18‐ to 59‐year‐old women and men in six Eastern and Southern African countries.^‡^

A total of 69,221 18‐ to 59‐year‐old women were sampled in the surveys and 65,607 (94.8%) women were eligible to participate in the survey. Among those who were eligible for the survey, 61,537 (93.8%) completed the interview, 56,698 (92.1%) provided a valid blood specimen and 56,333 (99.4%) answered the AUDIT‐C questions (Figure 1). A total of 59,528 18–59 y/o men were sampled in the surveys and 53,019 (89.1%) were eligible to participate.  Among those who were eligible, 44,662 (84.2%) completed the interview, 40,211 (90.0%) provided a valid blood specimen and 39,878 (99.2%) answered the AUDIT‐C questions (Figure 1). By country, Zambia had the highest proportion of HIV‐positive men who reported HAU (35.6%), and Namibia had the highest proportion of HIV‐positive women who reported HAU (16.0%) (Table 1).

**Table 1:** Hazardous alcohol use among HIV‐positive adults aged 18–59 years by selected demographic characteristics—six Population‐based HIV Impact Assessments, 2015–2017

	Hazardous alcohol use^a^			
	Male		Female	
Variable	Weighted % (95% CI)	Total^b^	Weighted % (95% CI)	Total^b^
Total	21.8 (20.4–23.3)	4444	6.6 (5.9–7.3)	9755
Age				
18–24	4.0 (1.9–6.1)	175	5.4 (3.6–7.2)	822
25–29	23.6 (18.9–28.3)	342	8.1 (6.1–10.0)	1321
30–39	27.3 (24.7–29.9)	1439	7.1 (6.0–8.3)	3596
40–59	19.5 (17.6–21.4)	2437	5.7 (4.6–6.8)	3795
Mean age	39.7 (38.7–40.0)	4444	36.0 (35.0–37.0)	9755
Country				
Eswatini	16.3 (12.9–19.7)	836	4.1 (3.1–5.1)	1878
Malawi	18.2 (15.2–21.2)	656	2.8 (1.7–3.9)	1451
Namibia	24.6 (20.3–28.8)	688	16.0 (14.1–17.9)	1588
Tanzania	15.8 (11.6–20.0)	485	5.8 (4.3–7.3)	1136
Zambia	35.6 (32.2–38.9)	743	13.0 (11.0–15.0)	1602
Zimbabwe	20.5 (17.8–23.2)	1036	3.9 (2.8–5.1)	2100
Place of residence				
Urban	27.2 (24.4–30.0)	1617	10.0 (8.7–11.3)	3925
Rural	18.5 (16.8–20.1)	2827	3.8 (3.2–4.5)	5830
Marital status				
Married/living together	21.4 (19.8–23.1)	3099	5.2 (4.3–6.0)	4781
Divorced	25.5 (19.3–31.7)	207	11.1 (8.6–13.7)	877
Separated	23.8 (18.6–28.9)	218	8.6 (5.8–11.5)	567
Widowed	22.2 (16.0–28.4)	165	4.9 (3.6–6.1)	1471
Never married	21.6 (17.4–25.9)	749	9.1 (7.2–11.0)	2021
Wealth quintile				
Lowest	20.1 (17.3–22.9)	615	1.9 (1.3–2.5)	1248
Second	19.5 (16.2–22.9)	708	4.1 (2.9–5.2)	1427
Middle	15.6 (13.1–18.1)	1001	5.3 (3.6–6.9)	2061
Fourth	25.3 (21.7–28.9)	1136	7.3 (5.9–8.7)	2545
Highest	28.1 (24.8–31.3)	984	11.5 (9.7–13.2)	2474

^a^ Defined as a pattern of alcohol consumption that increases the risk of harmful consequences for the user or others.

^b^ Unweighted.

### 3.1 Demographic factors

Among the 9755 women and 4444 men who tested HIV positive, 745 (6.6%) of women and 960 (21.8%) of men engaged in HAU (Table 1). Among HIV‐positive women, 405 (10.0%) who reported HAU resided in an urban area; 102 (11.1%) were divorced; 241 (11.5%) were in the highest wealth quintile; and 125 (8.1%) were aged 25–29 years (Table 1). The mean age of participants who engaged in HAU was 38 years (36 years among women and 40 years among men). Among HIV‐positive men who reported HAU, 411 (27.2%) lived in an urban area; 55 (25.5%) were divorced; 240 (28.1%) were in the highest wealth quintile; and 372 (27.3%) were aged 30–39 years (Table 1).

### 3.2 UNAIDS 90‐90‐90 targets

**Figure 2:**
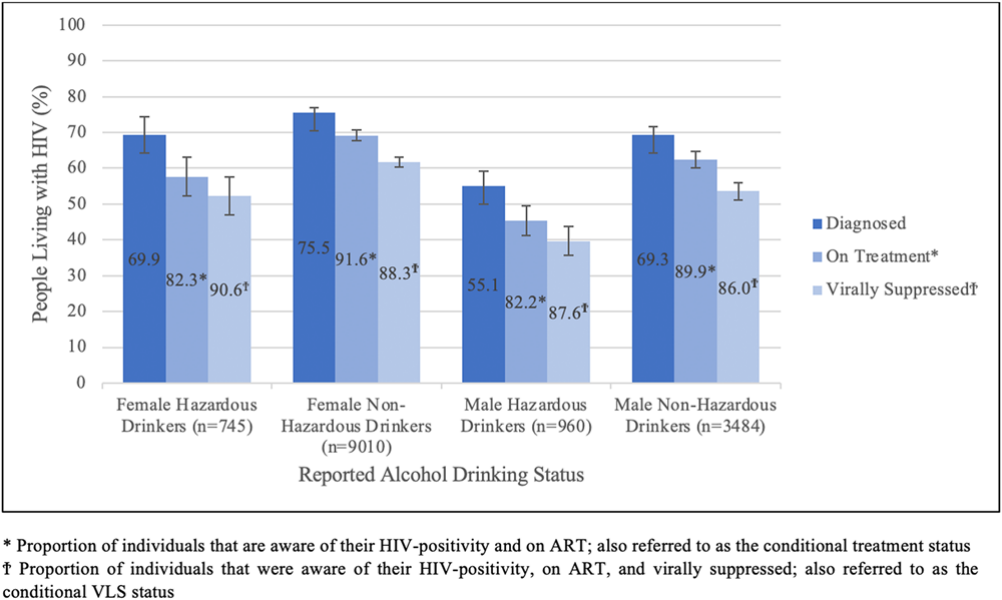
Weighted conditional prevalence estimates for the UNAIDS 90‐90‐90 targets by sex and hazardous drinking status—six Population‐based HIV Impact Assessments 2015–2017.

Of the 3324 women who reported HAU, 745 (19.1%) tested HIV positive. Among the 7986 men who reported HAU, 960 (9.9%) tested HIV positive (Table 2). Awareness was lower among those who reported HAU compared to those who did not report HAU, where 69.9% (95% CI: 64.8–75.1) of women and 55.1% (95% CI: 51.1–59.1) of men were aware of their HIV‐positive status (Figure 2). Among those who reported HAU and were aware of their HIV‐positive status, 82.3% (95% CI: 77.6–87.1) of women and 82.2% (95% CI: 78.3–86.1) of men were on ART (Figure 2). Among women who reported HAU, aware of their HIV status and on ART, 90.6% (95% CI: 87.5–93.7) were virally suppressed. For men in this same group, 87.6% (95% CI: 83.9–91.2) were virally suppressed (Figure 2).

**Table 2:** Weighted prevalence estimates and adjusted prevalence ratios for indicators among adults 18–59 years by sex and self‐reported alcohol use status—six Population‐based HIV Impact Assessments, 2015–2017

	Hazardous drinkers		Non‐hazardous drinkers		aPR^b^
Indicator	% (95% CI)	*n* ^a^	% (95% CI)	*n* ^a^	% (95% CI)
Female					
HIV positive	19.1 (17.2–21.0)	3324	11.4 (11.1–11.8)	53,009	1.31 (1.18–1.46)
Not on ART (aware of status)	17.7 (12.9–22.4)	566	8.4 (7.6–9.2)	7445	1.73 (1.26–2.37)
Unaware of HIV‐positive status	30.1 (24.9–35.2)	745	24.5 (23.1–25.1)	9010	1.22 (1.01–1.47)
No VLS (on ART)	9.4 (6.3–12.5)	486	10.7 (9.7–11.7)	6791	0.90 (0.61–1.32)
Not on ART (unconditional)	42.2 (37.0–47.8)	745	30.8 (29.4–32.2)	9010	1.33 (1.15–1.53)
No VLS (unconditional)	47.8 (42.5–53.1)	745	38.2 (36.9–39.6)	9010	1.22 (1.08–1.38)
Male	% (95% CI)	*n* ^a^	% (95% CI)	*n* ^a^	% (95% CI)
HIV positive	9.9 (9.1–10.8)	7986	6.9 (6.5–7.2)	31,892	0.94 (0.86–1.04)
Unaware of HIV‐positive status	44.9 (40.9–48.9)	960	30.7 (28.3–33.0)	3484	1.56 (1.39–1.76)
Not on ART (aware of status)	17.8 (13.9–21.7)	593	10.1 (8.7–11.5)	2652	1.72 (1.30–2.29)
No VLS (on ART)	12.4 (8.8–16.1)	492	14.0 (12.2–15.8)	2419	0.90 (0.63–1.29)
Not on ART (unconditional)	54.7 (50.6–58.8)	960	37.7 (35.4–40.0)	3484	1.51 (1.37–1.67)
No VLS (unconditional)	60.3 (56.4–64.3)	960	46.4 (44.1–48.8)	3484	1.34 (1.23–1.45)

Note: Unconditional ART status was defined as individuals who were not on ART regardless of being aware or unaware of their HIV status.

Unconditional VLS status was defined as individuals who did not have VLS regardless of being on or off ART.

Abbreviations: aPR, adjusted prevalence ratio; ART, antiretroviral therapy; CI, confidence interval; HIV, human immunodeficiency virus; VLS, viral load suppression.

^a^ Unweighted denominators of corresponding row percentages.

^b^ Adjusted for place of residence, global wealth quintile, age, country and marital status.

Table 2 shows that women who engaged in hazardous drinking were 31% more likely to be HIV positive compared to those who did not engage in HAU. Women who engaged in HAU were more likely to be unaware of their HIV‐positive status than those who did not engage in HAU (aPR = 1.22, 95% CI: 1.01–1.47). Among women who were aware of their HIV‐positive status, hazardous drinkers were significantly more likely not to be on ART (aPR = 1.73, 95% CI: 1.26–2.37) compared to women who did not engage in HAU. However, among women who were aware of their status and on ART, there was no difference in VLS by HAU (aPR = 0.90, 95% CI: 0.61–1.32). For unconditional ART and VLS targets, women living with HIV who reported HAU were more likely not to be on ART (aPR = 1.33, 95% CI: 1.15–1.53) and not to be virally suppressed (aPR = 1.22, 95% CI: 1.08–1.38) compared to those who did not report hazardous drinking.

Men who reported HAU were also found to be more likely to be unaware of their HIV‐positive status (aPR = 1.56, 95% CI 1.39–1.76). Men who engaged in HAU and were aware of their HIV‐positive status were also more likely not to be on ART (aPR = 1.72, 95% CI 1.30–2.29), compared to men who did not engage in HAU. There was no difference in the conditional VLS status in men by reported HAU (aPR = 0.90, 95% CI: 0.63–1.29). For unconditional ART and VLS targets, men living with HIV who reported HAU were more likely not to be on ART (aPR = 1.51, 95% CI: 1.37–1.67) and not to be virally suppressed (aPR = 1.34, 95% CI: 1.23–1.45) compared to those who did not report HAU (Table 2).

## 4 DISCUSSION

Among both men and women who engaged in HAU in this analysis, the largest gap in meeting the UNAIDS 90‐90‐90 targets was in the awareness of HIV‐positive status, where about half of men and one‐third of women did not know their HIV‐positive status. Due to the large gap in awareness among individuals with HAU, increased HIV testing strategies that target individuals at the highest risk, such as provider‐initiated testing and counselling (PITC), index partner testing and HIV self‐testing, may be recommended to quickly identify these individuals as opposed to more traditional strategies, such as voluntary testing and counselling [33, 34]. By identifying individuals who engage in HAU, interventions that provide more focused case management services can help to ensure successful linkage and retention in treatment, especially in resource‐limited settings. Current examples of these types of interventions in sub‐Saharan Africa include the electronic screening and brief intervention as well as the Common Elements Treatment Approach, both of which incorporate the full AUDIT screening tool and refer those at‐risk for alcohol use disorders (AUDs) to alcohol intervention modules [35, 36].

### 4.1 HIV prevention strategies

Venue‐based interventions at bars, nightclubs or other popular alcohol‐serving establishments provide opportunities for HIV prevention. A review investigating HIV prevention programmes in alcohol‐serving venues supported the implementation of multi‐level interventions, at the structural, social and individual levels, for HIV prevention and alcohol reduction strategies [37, 38]. These types of interventions can come in the form of recruiting individuals from alcohol‐serving venues for HIV testing and counselling, HIV health education and prevention strategies, alcohol reduction workshops, on‐site condom distribution and promotion of adherence to ART for HIV‐positive individuals in care. Our analysis adds to the growing body of evidence calling for alcohol reduction or methods of addressing hazardous drinking among PLHIV [37, 38].

### 4.2 HIV care cascade

Among those who were aware of their HIV‐positive status, both men and women who reported HAU did not meet the 90% conditional treatment target, suggesting gaps in treatment linkage and retention. This gap may signify a need for Screening, Brief Intervention and Referral to Treatment (SBIRT) among newly diagnosed HIV‐positive individuals and ART clients. A pilot study conducted in Namibia involving 787 participants found SBIRT to be feasible for identifying hazardous drinking behaviour as well as the participants having an overall beneficial experience [36]. Our results are similar to another population‐based study conducted in Kenya and Uganda, in which no association between alcohol use and VLS was found among those who were on ART [39]. This indicates more of a need to identify PLHIV and link to treatment as soon as possible. However, our analysis of VLS regardless of the awareness of HIV status shows that over half of HIV‐positive men and women who engage in HAU are not virally suppressed (Table 2). The low rate of VLS among these individuals may also be related to the overall lack of awareness of their HIV status, which in turn affects treatment and, therefore, VLS. The implications of high viral loads are an increased likelihood of sexual transmission of HIV, increased mortality and poor health outcomes; thus, it is imperative that these individuals are identified, retained on treatment and achieve VLS [20].

Our results show that both men and women who reported hazardous alcohol consumption did not meet the conditional ART target, alluding to breaks in care/treatment. Because of this, our findings suggest that further evaluation is warranted by clinicians with individuals who screen positive for HAU to provide supportive treatment and substance use counselling. In short, alcohol risk screening tools could be used as part of routine HIV programming for healthcare providers to incorporate alcohol reduction interventions to improve the overall HIV care and clinical management of individuals most at risk for AUDs and poor HIV outcomes.

Our findings are subject to several limitations. Due to the cross‐sectional nature of the PHIAs, causation cannot be determined and the element of temporality between HAU and HIV infection needs to be explored further. In addition, the questions used to categorize individuals as engaging in hazardous versus non‐HAU were based on self‐reported alcohol consumption questions, which is subject to desirability bias. This can result in an underestimation of HAU. More specifically, there has been evidence of women in sub‐Saharan Africa underreporting alcohol use due to the perceived negative stigma by their community, culture or religion [40, 41]. In addition, because not all individuals who were eligible for the survey chose to participate, non‐response bias may have influenced our prevalence estimates. It may be that those who chose to not participate were more likely to engage in HAU compared to those who participated in the survey. A final limitation is the data are from 2015 to 2017, which was due to the only PHIA datasets that were publicly available at the time of the analysis. The applicability of the findings may need to be adjusted to the current clinical and programmatic environment in each country. However, there is still strong evidence that hazardous drinking rates have largely remained unchanged [42]. Further, the challenges of identifying PLHIV engaged in hazardous drinking, and adherence to ART among PLHIV remains [42]. Because the next round of PHIA surveys includes the AUDIT‐C questions, our report provides baseline data for future studies to examine the evolving relationship between HAU and various HIV outcomes. A strength of the analysis is the correction of self‐reported awareness of HIV status and treatment status based on the detection of ARVs through laboratory testing, therefore, reducing the effect of desirability bias in reporting HIV awareness and treatment outcomes.

## 5 CONCLUSIONS

Our findings support the benefit of including alcohol screening tools as part of routine HIV testing and care and treatment programming in ESA. Referral for HIV testing may be needed for individuals who screen positive for hazardous drinking as part of PITC when individuals present for other conditions in healthcare settings. Findings from our analysis show that engagement in HAU among HIV‐positive adults was negatively associated with awareness of HIV‐positive status, being on ART and achieving VLS, which is a downstream effect of awareness and treatment status, among PLHIV across six PHIA surveys. Moreover, men and women who reported HAU did not meet the first two UNAIDS 90‐90‐90 targets, awareness of the HIV‐positive status and on ART, but did meet the VLS target. The largest gap in the HIV care continuum was awareness of HIV status followed by treatment status. Because a fifth of HIV‐positive men engaged in HAU, increased identification of HAU, testing, prevention and initiation into treatment may help improve the overall health of these individuals. If countries in ESA are to achieve HIV epidemic control by 2030, focus on underserved populations at risk for sub‐optimal HIV care, such as people who engage in hazardous drinking, is recommended.

## COMPETING INTERESTS

The authors state that they have no competing interests.

## AUTHORS’ CONTRIBUTIONS

GCC and CAW conducted the analysis and wrote the paper, with methodological support from EK, AJL, LAM, SM and ACV. Analytical support was provided by GH and RMB. EK, AJL, KEL, SSB, SYH, RS, GSM, JI, WMM, TC, AM, GM and CC revised the manuscript and provided critical input for intellectual content. ACV supervised the entirety of the manuscript. All authors reviewed and approved the manuscript.

## DISCLAIMER

The findings and conclusions in this manuscript are of the authors and do not necessarily represent the official position of the funding agencies.

## Data Availability

The data used in this manuscript are publicly available from the Population‐based HIV Impact Assessment (PHIA) Project website https://phia‐data.icap.columbia.edu/
